# Numerical Simulation on Thermal Stresses and Solidification Microstructure for Making Fiber-Reinforced Aluminum Matrix Composites

**DOI:** 10.3390/ma15124166

**Published:** 2022-06-12

**Authors:** Chenyang Xing, Reihaneh Etemadi, Krishna M. Pillai, Qian Wang, Bo Wang

**Affiliations:** 1 State Key Laboratory of Advanced Special Steel, Shanghai University, Shanghai 200072, China; xingcy136358@163.com (C.X.); wq06@shu.edu.cn (Q.W.); 2 School of Materials Science and Engineering, Shanghai University, Shanghai 200072, China; 3 Department of Mechanical Engineering, University of Wisconsin-Milwaukee, Milwaukee, WI 5322, USA; retemadi@uwm.edu (R.E.); krishna@uwm.edu (K.M.P.)

**Keywords:** fiber-reinforced, MMCs, numerical simulation, temperature field, microstructure

## Abstract

The fabrication of fiber-reinforced metal matrix composites (MMCs) mainly consists of two stages: infiltration and solidification, which have a significant influence on the properties of MMCs. The present study is primarily focused on the simulation of the solidification process and the effect of the active cooling of fibers with and without nickel coating for making the continuous carbon fiber-reinforced aluminum matrix composites. The thermomechanical finite element model was established to investigate the effects of different cooling conditions on the temperature profile and thermal stress distributions based on the simplified physical model. The predicted results of the temperature distribution agree well with the results of the references. Additionally, a three-dimensional cellular automata (CA) finite element (FE) model is used to simulate the microstructure evolution of the solidification process by using ProCAST software. The results show that adding a nickel coating can make the heat flux smaller in the melt, which is favorable for preventing debonding at the coating/fiber and alloy interface and obtaining a finer microstructure. In the presence of the nickel coating, the number of grains increases significantly, and the average grain size decreases, which can improve the properties of the resultant composite materials. Meanwhile, the predicting results also show that the interfaces of fiber–coating, fiber–melt, and coating–melt experience higher temperature gradients and thermal stresses. These results will lead to the phenomenon of stress concentration and interface failure. Thus, it was demonstrated that these simulation methods could be helpful for studying the solidification of fiber-reinforced MMCs and reducing the number of trial-and-error experiments.

## 1. Introduction

Metal matrix composites (MMCs) are usually composed of a metal or alloy as the continuous phase and whiskers or fibers of a reinforcing material as the second phase [1,2]. MMCs are widely used in developing materials for aerospace, electronics, and optical instruments due to their good mechanical properties, including low density, high Young’s modulus, high wear, and fatigue resistance. However, factors such as poor wettability, chemical reaction at the melt–fiber interface, and larger grain size and dendrite arm spacing during solidification processing tend to restrict the development of these materials for the industry [1,2,3,4,5,6,7,8].

The pressure infiltration process (PIP) is an established technique to manufacture MMCs where liquid metal or alloy is injected into a dry porous medium called the preform, made of reinforcing fibers, and later solidified to create the solid composite. Such fabrication of MMCs includes two stages: infiltration and solidification [1,5,9,10]. The liquid–metal infiltration process is a complicated flow and transport phenomenon, which can involve the preferential flow of liquid metal through larger pores of the preform, the mechanical deformation of the preform, and the solidification of liquid metal on coming in contact with cooled fibers and surfaces [11]. Transport phenomena during infiltration govern the temperature and solute distributions at and behind the infiltration front. These phenomena are often accompanied by other phenomena such as the segregation of alloying elements and chemical reactions. Finally, the solidification of the metal matrix occurs during and after the infiltration process, resulting in the final MMC part. In practice, all these phenomena simultaneously occur during the infiltration process.

The interface problem has been a core issue in manufacturing metal matrix composites, especially for active metals such as aluminum [12,13,14,15]. High infiltration temperature is a key factor in preparing carbon and aluminum (C/Al) composites. However, at this temperature, aluminum readily reacts with carbon to form a brittle phase of Al_4_C_3_ between carbon fibers and the aluminum matrix, which leads to the degradation of carbon fibers and consequently results in the deterioration of the mechanical properties of the composite and leads to its early failure under load [15]. High-resolution microfractography and transmission electron microscopy show that the mechanical behavior of the carbon-fiber-reinforced Al-based matrix composites is related to the presence of brittle interfacial phases [16]. The most common way to solve this problem is to coat the surface of the carbon fibers using the vapor deposition technique with nickel or copper [15]. This coating not only reduces the reaction at the fiber–melt interface, but also improves the wettability [17,18,19]. By using the nanoindentation technique, A. Urena et al. [19] investigated the interfacial mechanical properties of an AA6061 composite reinforced with short carbon fibers coated with copper and nickel films. The film coating on the carbon fiber surface was applied to control the interfacial reactivity of fibers with molten aluminum during the manufacture of the composite. The results showed that the copper coating produced by electroless increases the hardness and stiffness of the aluminum matrix, and nickel coatings decrease the hardness of the matrix close to the fibers and produce a high dispersion of stiffness values, especially in the own interface and at distances above 5 μm from the fibers. Improving the interfacial bonding between fibers and melt is one of the key factors in improving the properties of the fiber-reinforced composites [15,20]. The coating can play the adhesive role on the interface, leading to an improvement in the load transfer to the fibers. It has also been observed experimentally that the presence of carbon fibers alters the microstructure of the matrix alloy created during solidification. For example, Z.G. Liu et al. [21] studied the interface in the carbon fiber-reinforced Al–Cu alloy composites. The important feature observed in their experiments was that the microstructure of the Al–Cu matrix alloy was altered due to carbon fibers.

Nickel and copper are among the widely used coating materials. Although these metallic coatings can improve the wetting of carbon materials, the formation of intermetallic compounds or carbides will reduce the mechanical properties of the composites. This shortcoming should be controlled by optimizing the coatings’ thickness and the composites’ fabrication parameters. In the present study, the effect on the solidification of fibers with and without nickel coating was investigated. The effect of the brittle phases or transition phases formed at interfaces is ignored in the simulation study.

The solidification of MMCs is essentially a process of nucleation and the growth of crystals for base alloys. In an actual process, the grain growth is always accompanied by the phenomena of dendrite remelting and dendrite segregation. However, this effect is often ignored in the numerical simulation of the MMC solidification due to its complexity and little effect on the overall results. Many works of the simulation on infiltration and solidification processes for making MMCs have been done, but little study has been implemented on microstructure simulations, especially the nucleation and growth of crystals during the solidification process [22,23,24,25,26,27]. The main reason is that the presence of reinforcement materials in the metal matrix composites makes the solidification process more complicated. At present, the cellular automata (CA) method and phase-field method are two commonly used methods for simulating the microstructure evolution in solidification processes [28]. Although the phase-field method is accurate for the simulation of microstructure evolution due to its foundation on thermodynamics and physically-informed parameters, its overly complex principle, the need for more enormous computational resources, and the small computational domain hinder its further industrial application. In this study, the CA method is used to simulate the nucleation and growth of crystals in the solidification processes for making MMCs.

The microstructure development in MMCs is closely related to the temperature field, which can be changed by controlling the cooling rate. Lee et al. [27] studied the effect of the cooled fibers on the solidification microstructures based on numerical simulation and experimental observation. The results show that cooling the ends of the fibers changed the cooling curves (temperature fields) to lead to the nucleation of aluminum dendrites on the surface of the fibers. In the absence of such cooling, primary aluminum nucleated away from the surface of the fibers, depositing the last freezing eutectic at the interface. A faster cooling rate will result in higher temperature gradients and the development of a fine grain structure [27,29]. In addition, the number of nucleation sites increases significantly due to the presence of the reinforcement phase, which is favorable to the formation of a large number of fine crystals. Lelito et al. [29] developed a numerical micro–macro model based on the empirical nucleation law to predict the grain density in the Mg-based MMCs. The experimental and simulation results also show that the cooling curves and matrix grain densities were a function of heat-extraction rates, mass fraction, and the particle diameter of SiC. So, one possible way for such microstructure improvement is to extend the ends of the reinforcing phase to the outside of the casting mold and cool the ends of the fibers with a heat sink. Due to a faster heat extraction, the solidification time is reduced. This method is referred to as the thermal management of fibers, which can significantly change the nature of the interface and the surrounding matrix and, therefore, the properties of the composite. Such an active fiber cooling method has also been used by researchers [30,31] to prevent damage to the nickel coating during the infiltration process. Rohatgi et al. [32] used the squeeze infiltration process to synthesize an MMC of Al-2014 reinforced with nickel-coated carbon fibers. They used a modified version of a commercial squeeze-casting machine in which the ends of the carbon fibers were made to extend out on both sides of the mold, so they were cooled due to the lower ambient temperature, resulting in a higher heat-transfer rate from the system.

Although significant work has been done to model the metal infiltration and solidification processes seen during the manufacture of MMCs, relatively less research has been conducted on modeling the evolution of grain microstructure during the solidification process [22,32]. Our study uses the fiber-based active cooling method employed in previous studies [17,30,32]. These previous works have shown that the solidification microstructures of fiber-reinforced aluminum composites can be altered by cooling the ends of the fibers extending out of the mold. Based on this method, numerical simulations have been done for a simplified model to study the temperature profile around the fibers and stress distribution with coating. However, the simulations of the microstructure evolution involving the grain nucleation and growth are not considered, which is key to the final properties of the MMCs. In this article, the temperature profile, stress distribution, and microstructure evolution around the fibers during solidification are simulated using the commercial software ProcCAST^®^ of ESI Group. The effect of nickel coating on the solidification process is also studied. These results should be helpful in controlling and optimizing the solidification process witnessed during the making of MMCs.

## 2. Problem Description and Simulation Method

**Figure 1:**
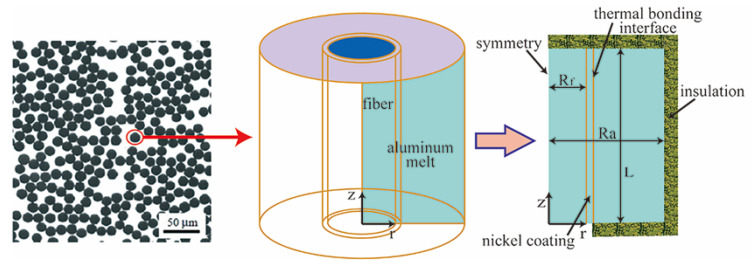
A schematic describing the 3D axisymmetric unit-cell for the carbon fiber/aluminum alloy MMC, as described in [17,30].

The conventional fiber preforms are made up of a large number of similar fiber units, as shown in Figure 1. For simplicity, a unit-cell of a cylindrical shape with a fiber and alumina melt is extracted as the calculation domain. Figure 1 presents the 3D-axisymmetric carbon fiber/aluminum model, in which the carbon fiber is located in the center and is wrapped in the aluminum melt. The physical model comes from the reference of Nguyen et al. [17,30]. This model assumes that a carbon fiber with or without coating is vertically oriented in the center and is surrounded by the Al alloy melt. Based on this model, this article discusses the temperature field and the thermal stresses and investigates the grain microstructure evolution, including the nucleation and growth process around the fiber and the trend of heat flux. The values of 1, 1, 0.2, and 0.05 units are set for radius (Ra), height (L), carbon fiber radius (Rf), and coating thickness parameters, respectively.

### 2.1. Mathematical Model

Considering only the postmold-fill scenario, we are going to model heat transfer, solidification, microstructure development, and stress estimation in a stationary pool of metal surrounding the fiber in the unit cell.

#### 2.1.1. Energy Equation

The energy conservation equation is solved to study the heat transfer and solidification phenomena in the postmold-fill process for making MMCs.

(1) $$\rho c_{p}\frac{\partial T}{\partial t}=\frac{\partial }{\partial x}\left(\lambda \frac{\partial T}{\partial x}\right)+\frac{\partial }{\partial y}\left(\lambda \frac{\partial T}{\partial y}\right)+\frac{\partial }{\partial z}\left(\lambda \frac{\partial T}{\partial z}\right)+Q$$

where ρ, $c_{p}$, *t*, and *T* are the density, the specific heat capacity, the time, and the temperature, respectively. $Q=\rho L\cdot \left(\partial f_{s}/\partial T\right)$, where L is the latent heat of melting and $f_{s}$ is the solid fraction.

#### 2.1.2. Thermal-Elastic-Plastic Model

The material begins to yield when the elastic deformation energy reaches a specified value under certain deformation conditions. The thermal–elastic–plastic model is adopted to simulate the deformation and thermal stress distribution during solidification for making MMCs.

The total strain increment includes thermal strain increment, elastic strain increment, and plastic strain increment. The effective stress is calculated by $\bar{\sigma }$.

The yield criterion follows the von Mises criterion:

(2) $$\bar{\sigma }=\frac{1}{\sqrt{2}}\sqrt{{\left(\sigma_{x}-\sigma_{y}\right)}^{2}+{\left(\sigma_{y}-\sigma_{z}\right)}^{2}+{\left(\sigma_{z}-\sigma_{x}\right)}^{2}}$$

where the $\sigma_{x}$, $\sigma_{y}$, and $\sigma_{z}$ are, respectively, the first, second, and third principal stresses.

#### 2.1.3. Nucleation and Growth Model

A three-dimensional cellular automaton–finite element (CAFE) model is used to simulate the microstructure evolution of the Al alloy composites. The continuous nucleation model, which employs the heterogeneous nucleation approach based on Gaussian distribution, is used. It should be mentioned that heterogeneous nucleation occurs in the bulk liquid, and the surface of the fiber or nickel coating is described by two distributions of nucleation sites which became active as undercooling increases. The continuous and nondiscrete distribution function is used to describe changes in grain density, which can be determined by Gaussian distribution [33,34] as

(3) $$\frac{dn}{d\left(\Delta T\right)}=\frac{n_{max}}{\sqrt{2\pi }\Delta T_{\sigma }}exp\left[-\frac{{\left(\Delta T-\Delta T_{max}\right)}^{2}}{2\Delta T_{\sigma }^{2}}\right]$$

where $\Delta T_{max}$, $\Delta T_{\sigma }$, and $n_{max}$ are the mean undercooling, the standard deviation, and the maximum density of nuclei, respectively.

In casting, the total undercooling of the dendrite tip is generally the sum of four contributions [33,34], as follows

(4) $$\Delta T=\Delta T_{c}+\Delta T_{t}+\Delta T_{k}+\Delta T_{r}$$

where $\Delta T_{c}$, $\Delta T_{t}$, $\Delta T_{k}$, and $\Delta T_{r}$ are the undercoolings contributions associated with solute diffusion, thermal diffusion, attachment kinetics, and solid–liquid interface curvature, respectively.

They are the undercooling contributions associated with solute diffusion, thermal diffusion, attachment kinetics, and solid–liquid interface curvature. The last three contributions are small for the solidification process for making MMCs, and the solute undercooling predominated.

During solidification, the constitutional supercooling and kinetic undercooling affect the dendrite growth. In general, constitutional supercooling plays a decisive role in the growth of the dendrite tip. Thus, its growth kinetics can be predicted effectively using KGT (Kurz–Givoanola–Trivedi) model [34]. Hence, the growth rate formula for the dendrite tip can be expressed as [33,34]

(5) $$\vartheta_{tip}=\alpha {\left(\Delta T\right)}^{2}+\beta {\left(\Delta T\right)}^{3}$$

where *α* and *β* are empirical constants.

### 2.2. Material Properties

The material properties of the fiber, the nickel coating, and the melt used in the analysis are presented in Table 1. Values of some primary parameters used in the microstructure simulation are shown in Table 2. (These parameters on microstructure growth are mainly taken from the ProCast manual.) Coefficients of the growth kinetics are calculated by the module in the ProCast2009 software. The calculated results are quite consistent with the values available in the literature.

### 2.3. Initial and Boundary Conditions

The adiabatic thermal boundary condition is applied to the outer surfaces of the model except at the bottom of the carbon fiber and coating. The carbon fiber’s bottom surface (z = 0) is given a constant temperature of 25 °C. The initial temperature of the fiber and the aluminum melt is considered to be 639 °C. It is assumed that the melt–fiber interface is ideal in terms of contact conditions, while the inner surface of the carbon fiber and coating are in complete contact, and the formula for the coefficient of the interface between coating and melt can be expressed as

(6) $$\lambda {\frac{\partial T}{\partial n}|}_{w}=h_{i}\left(T_{w1}-T_{w2}\right)$$

where $\lambda {\frac{\partial T}{\partial n}|}_{w}$ and $h_{i}$ are the normal temperature-gradient-driven heat flux at the boundary (with λ being the thermal conductivity) and the boundary heat transfer coefficient, respectively. *T_w1_* and *T_w2_* represent the surface temperatures of the coating and melt, respectively.

For the thermal-stress analysis, a zero-displacement boundary condition is employed on the outer surface of the mesh model, which prevents deformation in the normal direction, but allows displacement in the tangential direction. Thermal stresses are calculated from the temperature field at any given time. The melt–fiber or melt–coating interfaces are assigned as nucleation sites for the microstructure simulation.

### 2.4. Numerical Solution

In this paper, the finite element method (FEM) is used for the numerical solution of the problem. The calculation domain should be simplified as a microunit (cylinder), which size is the one unit of height and one unit of diameter, as shown in Figure 1.

In the simulation coupling temperature and stress, though the temperature field is calculated at each time step, the coupled thermal stress field begins to be calculated when the solidification fraction of the melt reaches 50%. The iterative procedure is continued until the values at each node converge. The calculating procedure is stopped when the liquid metal is completely solidified.

In the solidification microstructure simulation, the cellular automaton (CA) finite element (FE) model is used to simulate the microstructure of Al alloy composites. In the simulation domain, the larger mesh is used to simulate the temperature and enthalpy defined at each node using the energy equation. Subsequently, the cell meshes with smaller size are used for microstructure calculations interactively by the CA method within the temperature range calculated by the FE method at each macrotime step. The nucleation and the dendritic-growth computations within the CA method are two significant components of the microstructure simulation described in Section 2.1.3.

**Figure 2:**
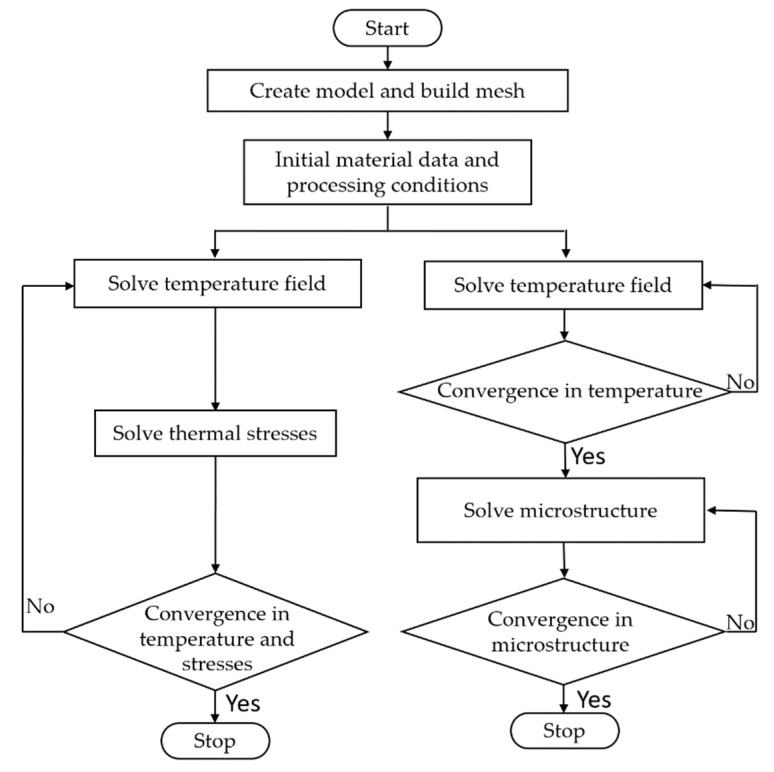
Flow chart of the numerical solution procedure adapted for modeling the solidification process of MMCs.

The flow charts of the numerical solution procedure developed for this simulation are shown in Figure 2. The present study is proposed to compute changes in the temperature and thermal stress fields with time and to determine the microstructure evolution during the solidification process of the Al alloy considered. The thermal stress simulation and thermal grain structure simulation are calculated using the commercial software ProCast in our study. The solution domain is discretized into 174,661 nodes, the mesh grid size is about $2.5\times 10^{-3}\;\mathrm{mm}$, and the conservation equations are solved at each node of the elements. Such a procedure is repeated iteratively until convergence to the correct solution is obtained. The minimum time-step is about 0.001 s, and the convergence criteria for energy equation is 10^−6^. During the presentation of the results, some variables involved in the governing equations are rendered dimensionless, including geometry, temperature, and time equations [17,30]:

(7) $$\;\;\bar{r}=\frac{r}{R_{a}},\;\bar{z}=\frac{z}{R_{a}},\;\;{\bar{R}}_{f}=\frac{R_{f}}{R_{a}}$$

(8) $$AR=\frac{L}{R_{a}},\theta_{m}=\frac{T_{m}-T_{0}\;}{T_{i}-T_{0}},F_{o}=\frac{t\alpha }{R_{a}^{2}},\;\;\bar{HF}=\frac{HF}{-k_{a}\left(T_{i}-T_{0}\right)/R_{a}}$$

where *r* is the radial distance, $k_{a}$ is the thermal conductivity of the alloy, and *T_m_*, *T_0_*, and *T_i_* is the temperature of the alloy, the cooling temperature, and the initial temperature, respectively. The symbol t represents the time, and a is the thermal expansion coefficient. The symbol with a bar represents the dimensionless parameters. AR is the aspect ratio of the mold, the dimensionless temperature of the alloy, the dimensionless time, and the dimensionless heat flux.

## 3. Results and Discussion

In the present study, the aluminum alloy melt has been pressurized and completely infiltrated into the mold packed with the fiber preform. The infiltration flow and the concomitant interface reactions are neglected. However, the effects of the cooling conditions and the coating on the temperature, thermal stress, and microstructure are considered in this analysis.

**Figure 3:**
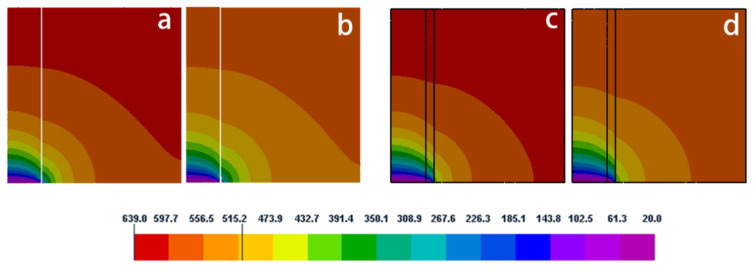
Temperature profile in axial section of the model without coating (**a**,**b**) and with coating (**c**,**d**) at different dimensionless times (**a**,**c**) Fo = 0.156, (**b**,**d**) Fo = 0.234.

Figure 3 compares temporal changes in the temperature profile obtained from the axisymmetric model with and without nickel coating. The entire heat is extracted from the bottom of the extension fiber, so the low-temperature region is mainly concentrated in the lower part of the model. Meanwhile, the effect of the active cooling leads to a large temperature gradient near the fiber end. The nickel coating can act as a thermal barrier layer and make the melt’s temperature gradient steeper near the coating–melt interface. As we shall see later, this effect will result in a finer grain structure using nickel coating (Figures 14 and 15).

**Figure 4:**
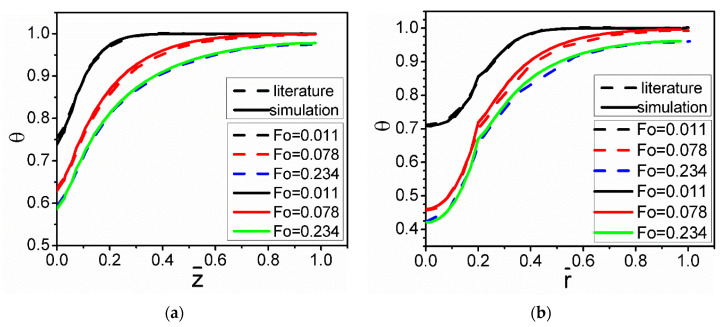
Comparison of the results from the present simulation with that reported in the literature [30] on the evolution of temperature without the nickel coating: (**a**) $\;\bar{r}$ = 0.25, (**b**) $\;\bar{z}$ = 0.1.

The present simulation was verified with the results reported by Nguyen et al. [30] for the numerical simulation using a 2-D model. Figure 4 compares the temperature distribution for the same conditions. The results from the present 3-D simulation show reasonably good quantitative agreement with the results obtained by Nguyen. From this figure, it can also be seen that the temperature gradient of the melt decreases with increases in the axial and radial distances.

**Figure 5:**
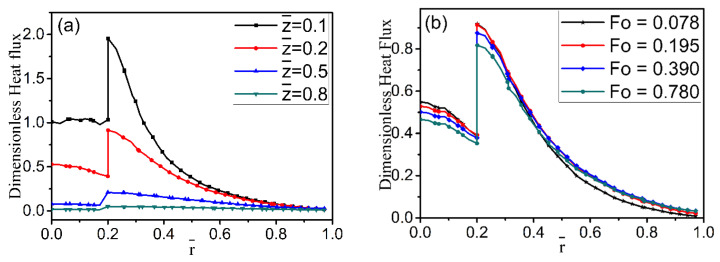
Effect of different distances on dimensionless heat flux without the nickel coating: (**a**) at Fo = 0.195; (**b**) at $\;\bar{z}$ = 0.2.

**Figure 6:**
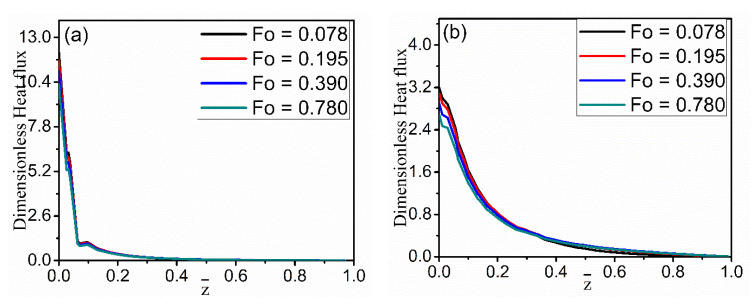
Changes in the dimensionless heat flux (without the nickel coating) along the fiber-axis direction at (**a**) $\;\bar{r}$ = 0.19, (**b**) $\;\bar{r}$ = 0.26.

Dimensionless heat flux as a function of radial location is studied for four axial-location cases and different time cases in Figure 5. It can be seen that the heat flux within the fiber changes little along the radial direction, but the heat flux in the melt declines sharply. There is a discontinuity in the heat flux profile due to a sharp change in the thermal properties across the fiber and melt interface. It is a potential site for stress concentration. Figure 5a also illustrates that the heat flux has an obvious decreasing trend away from the cooling end along the z-direction. Figure 5b shows the changes in the nondimensional heat flux along the axial (z) direction at the different time instances. In Figure 6, the heat flux obviously decreases along the axial direction as the radial distance increases from r = 0.19 to r = 0.26. However, there are differences in the two figures, because r = 0.19 is located inside the fiber and is near the cold fiber-end, while r = 0.26 is located in the melt. Inside the fiber, the decline in heat flux is much steeper than that in the melt, which may be attributed to differences in thermal conductivity in the two regions (Table 1).

**Figure 7:**
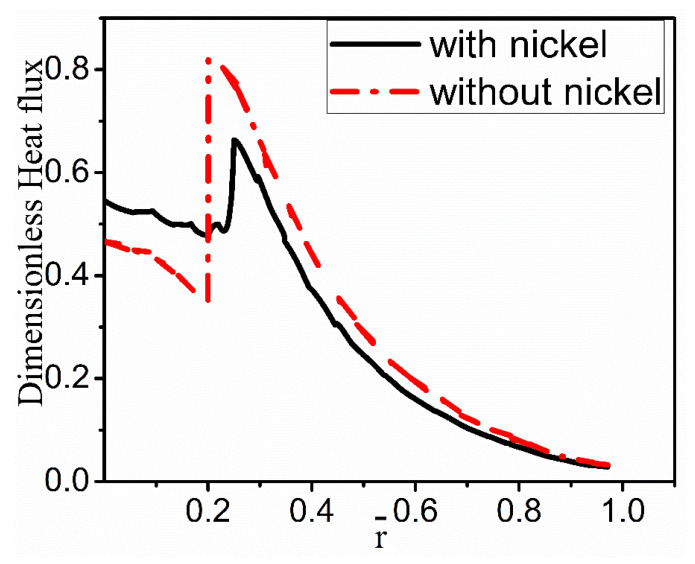
Dimensionless heat flux with and without nickel coating at $\;\bar{z}$ = 0.1.

Figure 7 compares the heat flux with and without nickel coating along the radial direction at the given z-direction location. It shows that the heat flux with nickel coating is less jagged than without nickel coating. The heat fluxes have a slight decline on the side of the fiber, then the expected increase near the interface, and then the smooth decrease on the melt side. Compared with the coating case, the increase in the heat flux is sharper near the interface for the no-coating case. This abrupt change is expected to produce a severe thermal stress concentration at the interface to result in the debonding of coating/fiber and alloy.

The thermal stress analysis is very effective for predicting stress concentrations, the resulting hot cracks, etc. The thermal stress is caused by temperature gradients in the fiber/alloy system. Residual stresses usually lead to thermal deformations in the material. Poor properties of metal matrix composites are often the result of undesirable heat distortions. Additionally, if the coating is considered in the model, the displacement caused by thermal deformation will become more complicated due to the different thermal expansion coefficients of the coating. Furthermore, nickel coating also has the function of transferring and bearing stresses.

**Figure 8:**
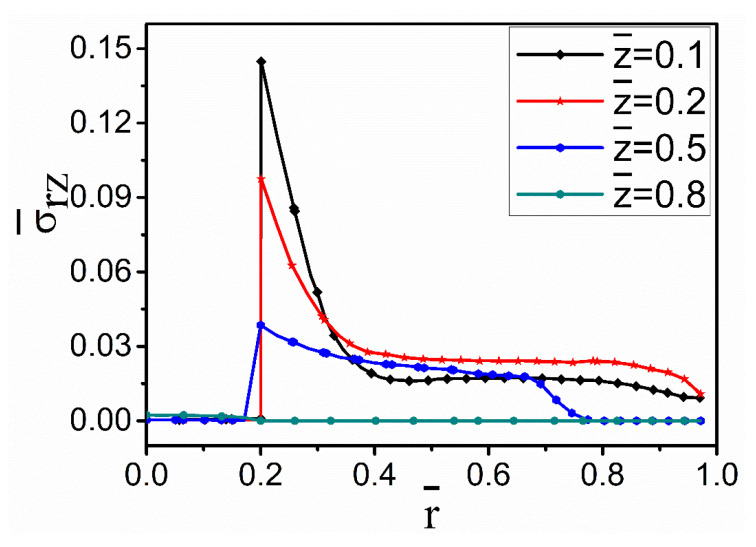
Variation in von Mises stress along the radial direction for the no-nickel-coating case at dimensionless time Fo = 0.195.

The stress concentration exists at the interface due to higher von Mises stress. As shown in Figure 8, there is a sharp decrease in the stress on both sides of the fiber–melt interface. On the side of the fiber, the thermal stresses are relatively stable; however, it declines sharply on the melt side. It is also clear that the stress ‘spikes’ decrease in intensity as one moves away from the cooled part of the fiber at =0. The spikes are correlated with the temperature gradients shown in Figure 3.

**Figure 9:**
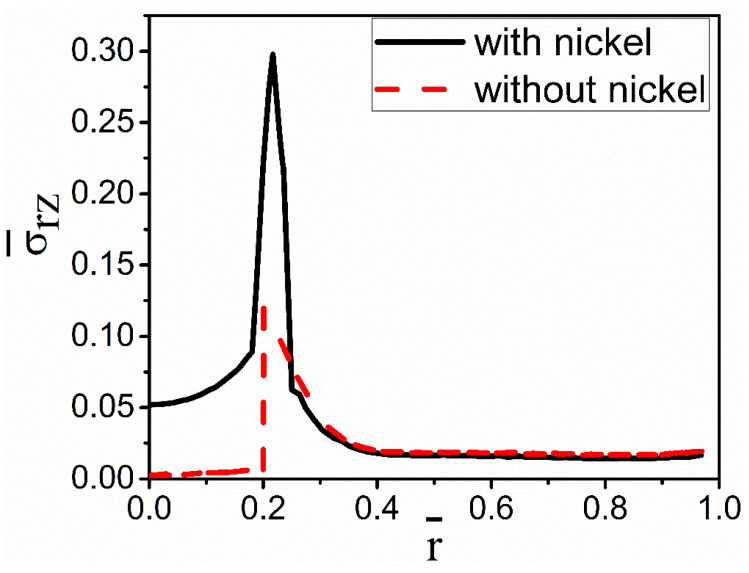
Radial changes in von Mises stress with and without nickel coating $\;\bar{z}$ = 0.1.

Figure 9 compares the resultant von Mises stresses with and without nickel coating. It can be seen that von Mises stress at the fiber–coating interface is much higher than at the fiber–melt interface without the coating. A close observation of this result reveals that nickel coating can cause the residual stress to increase significantly, and the stress in the fiber is higher than that in the melt. This higher stress may result in debonding of the coating. So, it is necessary to effectively control the cooling rate to protect against coating failure [36].

**Figure 10:**
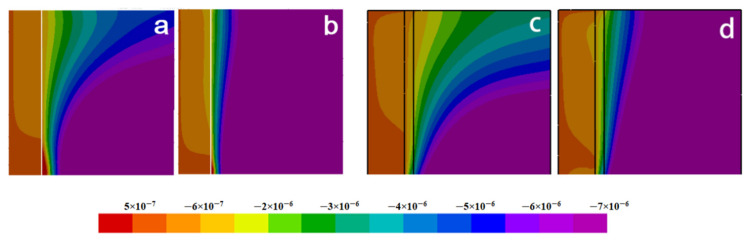
Distributions of radial deformation: (**a**) Fo = 0.156, without coating; (**b**) Fo = 0.234, without coating; (**c**) Fo = 0.156, with coating; (**d**) Fo = 0.234, with coating.

**Figure 11:**
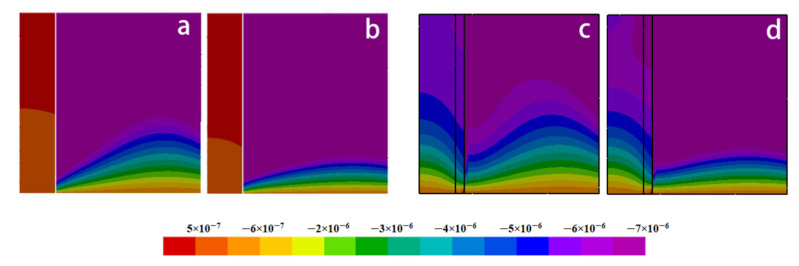
Distributions of axial deformation: (**a**) Fo = 0.156, without coating; (**b**) Fo = 0.234, without coating; (**c**) Fo = 0.156, with coating; (**d**) Fo = 0.234, with coating.

Figure 10 and Figure 11 illustrate the radial and axial deformation profiles at different cooling times. It can be seen that the extent of deformation, especially the axial one, in the fiber side with the nickel coating is far greater than that without the coating. However, the deformations are much smaller in the melt.

**Figure 12:**
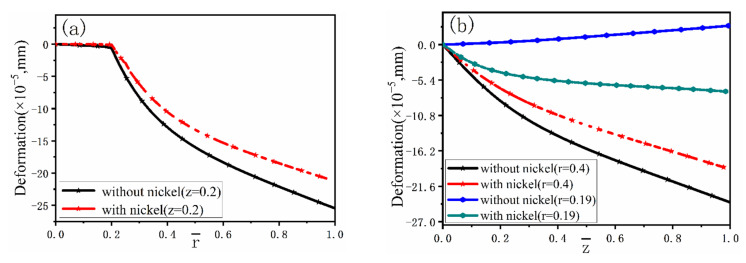
Deformation with and without nickel coating: (**a**) at $\;\bar{z}$ = 0.2, (**b**) at $\;\bar{r}$ = 0.19 and $\;\bar{r}$ = 0.4.

Figure 12 describes in detail comparison between the distributions of deformation with and without nickel coating. It can be found that the deformation displacement of the model with nickel coating is smaller than that without coating in the radial direction at $\;\bar{z}$= 0.2. At the same time, there is little change in displacement at the fiber side. Figure 12b, $\;\bar{r}$ = 0.19 reveals a completely different deformation of the model with and without nickel coating. The main reason is the different thermal expansion coefficient values for fiber, coating, and melt. The carbon fiber shows volume expansion with decreasing temperature, while the volume shrinks due to the positive thermal expansion coefficient of nickel coating and melt. In addition, the absolute value of the thermal expansion coefficient of the melt and the nickel coating is far greater than that of the fiber. So, the expansion process of the fiber is blocked and forced to move in the opposite direction during the cooling process. Figure 12b, $\;\bar{r}\;$= 0.4 presents that the deformation displacement of the model with nickel coating is smaller than without coating.

The nucleation and growth of grains during solidification is a key factor in determining the grain size and achieving the desirable properties of MMCs. It is not only related to the material itself but also closely connected with the cooling rate. Experimental investigations show that the grain size of the composites is often smaller than that of the unreinforced alloy under identical solidification conditions. Solute diffusion is impeded during growth due to the barrier effects of the reinforcement. Therefore, the delayed growth from the melt gives additional time for the formation of nuclei, which can yield a refined structure [27,28]. Microstructures in fiber-reinforced MMCs can be modulated in a predetermined manner by controlling interfiber spacing and cooling rate [28]. In the present study, the effects of cooling conditions and coating on grain microstructure were studied using the CAFÉ model. The cooling condition was controlled by varying the degree of cooling of the ends of fibers with water cooling: 25 °C and dry ice–acetone mixture: −78 °C.

**Figure 13:**
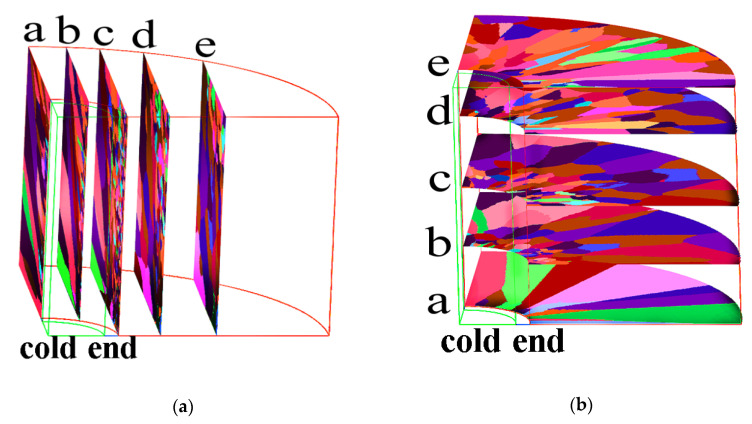
Microstructure predicted at the different sections: (**a**) axial sections, (**b**) radial sections.

**Figure 14:**
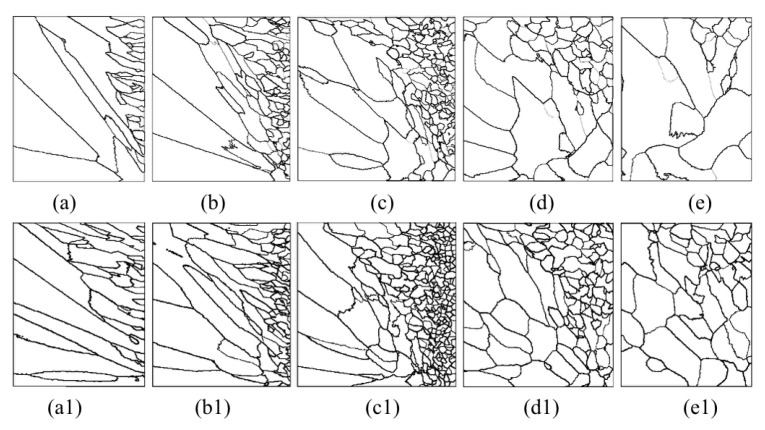
Microstructure morphology predicted of different axial sections with the cooling temperature of 25 °C (**a**–**e**) without nickel coating; (**a1**–**e1**) with nickel coating.

**Figure 15:**
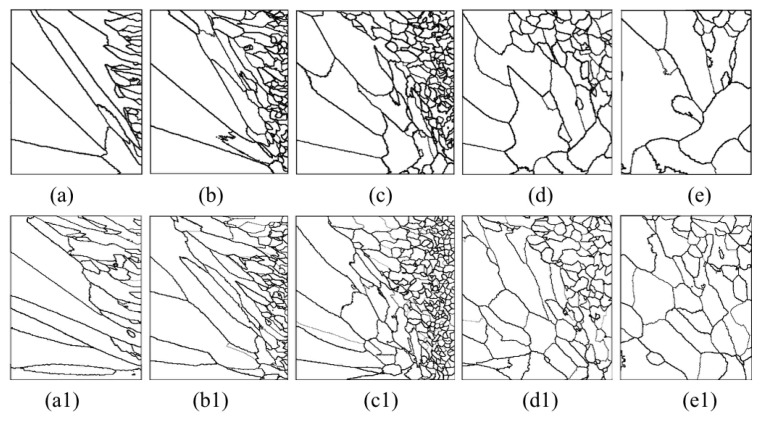
Microstructure morphology predicted of different axial sections with the cooling temperature of −78 °C (**a**–**e**) without nickel coating; (**a1**–**e1**) with nickel coating.

**Figure 16:**
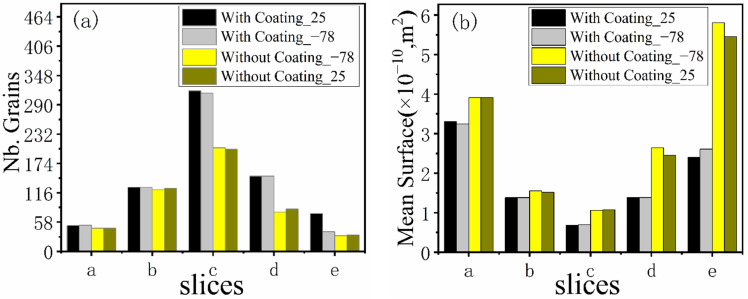
Number grains (**a**) and mean surface (**b**) of different axial sections.

Figure 13 shows a series of axial and radial slices of grain microstructure predicted. Figure 13, Figure 14 and Figure 15 present the simulation results of the grain microstructure profile of axial directions with and without coating under different cooling conditions. The higher temperature gradient is obviously affecting the microstructure close to the cold end. The grain growth near the nucleation surface is restrained and forms finer equiaxial crystals due to chilling. However, grains tend to form coarse columnar crystals away from the chilling layer, as shown in Figure 14(a–c,a1–c1) and Figure 15(a–c,a1–c1). The microstructure away from the fiber tends to form coarse equiaxed grains due to the lower thermal gradient away from the cold end, as shown in Figure 14(d,e,d1,e1) and Figure 15(d,e,d1,e1). Compared to the simulated results with and without nickel coating, it can be found from Figure 14(e,e1) and Figure 15(e,e1) that the number of grains increases significantly in the presence of the nickel coating away from the fiber. The size of grains also reduces relatively, as observed in Figure 14 and Figure 15. The main feature is that the nickel coating can act as a thermal resistance layer, making the temperature profiles near interfaces uniform and further reducing the temperature gradient, obtaining the fine grain structure. Similarly, this argument can be demonstrated in Figure 16. A close observation of Figure 16 reveals that as the location of the slice is far away from the carbon fiber (from (a) to (e)), the number of grains firstly increases and then decreases, the mean surface of grain firstly decreases and then increases. Moreover, it can be seen that there is a small difference in the number of grains and the average grain area at different cooling temperatures.

**Figure 17:**
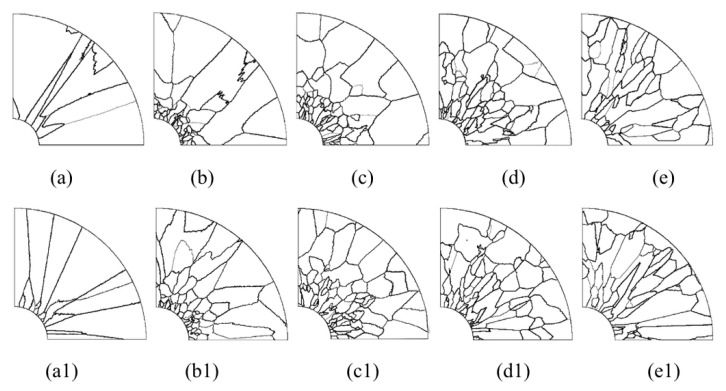
Microstructure morphology predicted of different radial sections with the cooling temperature of 25 °C (**a**–**e**) without nickel coating; (**a1**–**e1**) with nickel coating.

**Figure 18:**
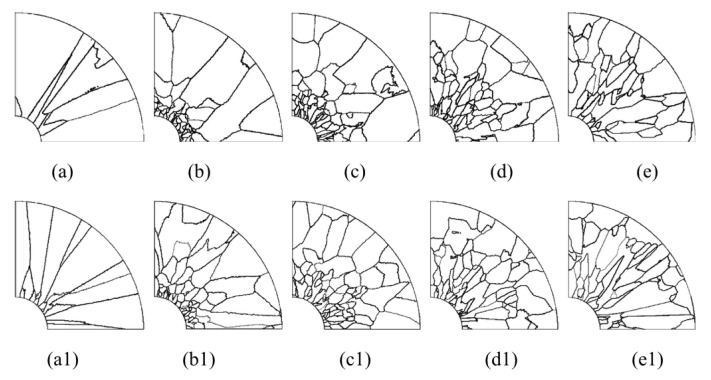
Microstructure morphology predicted of different radial sections with the cooling temperature of −78 °C (**a**–**e**) without nickel coating; (**a1**–**e1**) with nickel coating.

The microstructure morphology of the different radial sections is presented in Figure 17 and Figure 18. When slices are away from the cold end (from slice (a) to slice (e), as shown in the right picture of Figure 13), the grains will gradually become equiaxed grains at the edge of the slice. On the contrary, the grains will develop columnar crystals at the center of the section (near the carbon fiber). The reason for this situation is that the crystal growth direction is always in the opposite direction of heat flow.

**Figure 19:**
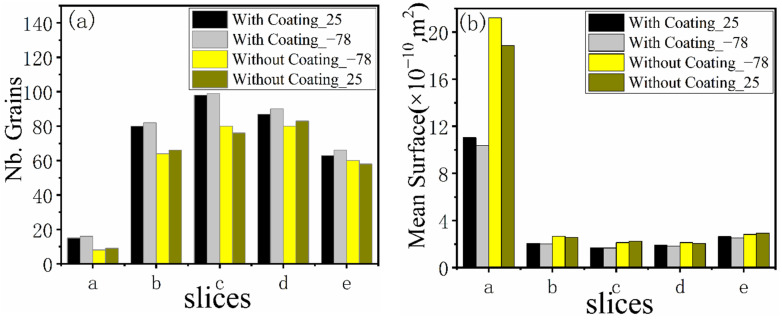
Number grains (**a**) and mean surface (**b**) of different radial sections.

Comparing the simulated microstructure of radial section with and without coating shows that the number of grains increases significantly, and the grains’ mean surface slightly decreases when using the nickel coating, which can be proved via Figure 19. Furthermore, Figure 19 also shows that the number of grains increases firstly and then decreases, and the mean surface presents the opposite trend with the increasing distance from the cold end. In conclusion, the microstructure is obviously refined, which is beneficial for improving the properties of metal matrix composites when the fiber is wrapped with nickel coating and the extended fiber cooling method.

**Figure 20:**
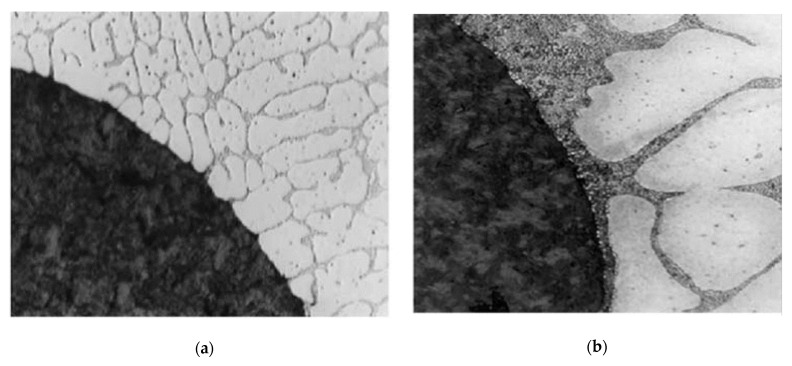
Solidifying microstructure of thermally managed Al-9% Cu alloy composite (**a**) with external cooling of graphite rod extending out of the melt and (**b**) without external cooling of the graphite rod [27].

An earlier study by Rohatgi [37] and Lee [27] revealed that cooling the extended ends of the reinforcement results in finer microstructures in the matrix and changes the nature of the interface. Figure 20 demonstrates the effect of fiber cooling on the matrix microstructure of an aluminum/carbon fiber composite, in which the carbon fibers were chilled outside of the mold [27]. In this case, very fine-sized grains were in contact with the surfaces of graphite fibers. The same results also can be observed from Figure 13, Figure 17, and Figure 18. The predicted results of solidification microstructure agreed well with these experimental results. So, it is demonstrated that the cooling of the extent of the fibers during making MMCs can be useful for producing the finer matrix microstructures around fibers and improving the properties of MMCs. However, suitable cooling conditions need to be designed and optimized in future works.

## 4. Conclusions

This paper mainly focuses on the numerical simulation of the temperature field, thermal stress, and microstructure of the fiber-reinforced metal matrix composites. Two physical models with and without nickel coating are analyzed in detail. At the same time, the effects of cooling rate on the resultant microstructure are also investigated by changing the fiber end cooling temperature. The following important conclusions are drawn from this study:

1. Based on a modified infiltration process by Nguyen et al., the effect of active cooling conditions on temperature distribution was simulated. The predicted results of temperature evolution agreed well with the reported results.
2. The distribution of heat flux has a significant influence on the microstructure and thermal stress. The heat flux trend is gradually evolving from the top of the model to the bottom of the fiber due to active cooling through carbon fiber. On the side of the fiber, the heat flux changes smoothly, while it varies drastically at the melt side. Comparing analysis results of the heat flux with and without nickel coating reveals that it is smoother and smaller in the Ni-coating model, which is favorable for preventing debonding at the interface of coating/fiber and alloy and obtaining the finer grains.
3. The predicted results of the thermal stress show that there is high thermal stress on the interfaces of fiber–coating, coating–melt, and fiber–melt. These places tend to cause stress concentration. On the one hand, it is easy to generate microcracks in these locations, resulting in interface failure; on the other hand, it tends to lead to debonding of the coating.
4. The formation and growth of grains are closely related to the temperature field. The heat is only dissipated from the bottom of the fiber. Therefore, the dendrites obliquely grew along with the model from the lower part of the fiber. The number of grains near the nucleation is more than that of the other places due to the effect of chilling. We also can see that the microstructure is significantly refined, and then the properties of metal matrix composites can be improved when the fiber is wrapped by nickel coating.

## Figures and Tables

**Table 1:** Material parameters gleaned from [35] and used in ProCast’s CAFE simulation.

Property	Carbon Fiber	Nickel	Al-2014
Thermal conductivity (W/m C)	54	60.7	193
Specific heat capacity (J/kg K)	921	460	880
Density (kg/m^3^)	1800	8880	2800
Thermal expansion coefficient (m/m °C)	−10^−7^	13 × 10^−6^	23 × 10^−6^
Young’s modulus (GPa)	217	207	71
Poisson’s ratio	0.3	0.31	0.33

**Table 2:** The in-built parameters used in ProCast’s CAFE simulation.

Property		Value
a2(First coefficient of the growth kinetics)		4.7 × 10^−6^
a3(Second coefficient of the growth kinetics)		2.5 × 10^−7^
Nucleation parameters in the bulk of the liquid (Gaussian distribution)	DTm (Average undercooling)	2.5
	DTs (Standard deviation)	1
	Nmax (Maximum number of nuclei)	7 × 10^10^
Nucleation parameters at the surface (Gaussian distribution)	dTm (Average undercooling)	0.5
	dTs (Standard deviation)	0.1
	Gmax (Maximum number of nuclei)	5.0 × 10^10^

## Data Availability

Not applicable.
